# The Self-Assembled Behavior of DNA Bases on the Interface

**DOI:** 10.3390/ijms15021901

**Published:** 2014-01-27

**Authors:** Lei Liu, Dan Xia, Lasse H. Klausen, Mingdong Dong

**Affiliations:** 1 Institute for Advanced Materials, Jiangsu University, 301 Xuefu Road, Jiangsu 212013, China; E-Mail: liu@ujs.edu.cn; 2 Interdisciplinary nanoscience Center (iNANO), Gustav Wieds vej 14, DK-8000 Aarhus C, Denmark; E-Mails: xiadan@inano.au.dk (D.X.); lassehyldgaard@inano.au.dk (L.H.K.)

**Keywords:** DNA base, self-assembly, interface chemistry, scanning tunneling microscopy

## Abstract

A successful example of self-assembly in a biological system is that DNA can be an excellent agent to self-assemble into desirable two and three-dimensional nanostructures in a well-ordered manner by specific hydrogen bonding interactions between the DNA bases. The self-assembly of DNA bases have played a significant role in constructing the hierarchical nanostructures. In this review article we will introduce the study of nucleic acid base self-assembly by scanning tunneling microscopy (STM) at vacuum and ambient condition (the liquid/solid interface), respectively. From the ideal condition to a more realistic environment, the self-assembled behaviors of DNA bases are introduced. In a vacuum system, the energetic advantages will dominate the assembly formation of DNA bases, while at ambient condition, more factors such as conformational freedom and the biochemical environment will be considered. Therefore, the assemblies of DNA bases at ambient condition are different from the ones obtained under vacuum. We present the ordered nanostructures formed by DNA bases at both vacuum and ambient condition. To construct and tailor the nanostructure through the interaction between DNA bases, it is important to understand the assembly behavior and features of DNA bases and their derivatives at ambient condition. The utilization of STM offers the advantage of investigating DNA base self-assembly with sub-molecular level resolution at the surface.

## 1. Introduction

In biological systems, the hierarchical structures of many biomolecules including DNA, peptide and protein are ascribed to self-assembly from molecular level to nanoscale. Among these, DNA is an ideal molecule capable of forming target-assembling structures based on the specificity of the base paring. In recent years, the complementary base pairing resulting from specific hydrogen bonds has been utilized to create desirable two-dimensional (2D) [1,2] or even three-dimensional (3D) nanostructures [3] in a well-controlled manner. It therefore seems obvious that the self-assembly of DNA bases plays a significant role in constructing hierarchical nanostructures. According to the molecular structure of DNA described by Watson and Crick around sixty years ago, a simple set of base-pairing rules was proposed for the four nucleic acid (NA) bases, guanine (G), cytosine (C), adenine (A) and thymine (T). However, it is significant to note that the NA bases are not exclusive in their binding behaviors. For example, at least 28 possible base-pairing motifs exist involving the hydrogen bonds formed between the four natural bases [4]. These NA base-binding modes can also play an important role in the NA self-assembly process and even in biological system. In addition, these single NA bases are capable of forming 1D or 2D supramolecular nanostructures when deposited onto various surfaces, as investigated in ultra-high vacuum (UHV) system. To understand the DNA replication mechanism in further detail it is reasonable to simplify the study by choosing the interaction of the pure bases as a model system. Here, we provide a tutorial on the study of self-assembly of nucleic acid (NA) bases based on hydrogen bonds by means of scanning tunneling microscopy (STM). The novel assembling structures of pure NA bases and of complementary bases will be introduced at vacuum and ambient condition, from ideal conditions to the more realistic environment, respectively. The self-assembly of NA bases into a diversity of nanopatterns on the surface, through intermolecular hydrogen bonding at different conditions will be presented. Finally, some prospective applications for the construction of self-assembled supramolecular nanostructures via base paring interactions are presented. The specific hydrogen bonding interactions between DNA bases involves multiple self-assembly systems including molecular recognition and assembled structure construction, which may be utilized in a variety of applications, such as the diagnosis of specific biomolecules related to diseases, and in nanomaterial construction.

## 2. Nanostructure Assembly in Two Dimensions (2D)

The DNA double helical structure is stabilized mainly due to the predominant hydrogen bonding between NA bases [5]. Besides the obvious chemical and electronic properties difference in NA bases, there is not a huge structural difference between them. In addition, it is a significant challenge to understand the mechanisms behind the assembly of NA bases and their complexes with amino acids, proteins and more complicated biological systems. It is therefore important to investigate self-assemblies of NA bases as a means of exploring the inter- and intra-molecular hydrogen bonding properties between them. Furthermore, complementary base pairing is applied in the recognition processes mediated by hydrogen bonding. The self-complementary interactions between NA bases have been observed in DNA molecules [6] and solid supports [7–9]. Recently, a diversity of binding possibilities between NA base dimers was well explored by theoretical calculations [10–12]. The dimer formation is the key component in building two-dimensional (2D) supramolecular structures on surfaces and it also reveals the diversity of interaction mechanisms between NA bases. STM is a powerful technique with high structural resolution capable of revealing that NA bases can self-assemble into a diversity of structures on a variety of surfaces under variable conditions [13–18]. STM studies of NA bases in vacuum systems allow the basic interactions and assembled behaviors of NA bases. Further study at the liquid/solid interface have the advantage that the liquid can, in principle, provide molecules with a certain degree of freedom during the physisorption process and thus a more biochemical environment may be taken into account. Moreover, the study of the self-assembly of NA bases by STM could also provide a better understanding of the forces necessary for creating 2D patterns on surfaces. In this section, we present the 2D supramolecular structures based on individual NA bases (Guanine, Cytosine, Adenine, Thymine) and complementary bases at vacuum system and ambient condition, respectively.

### 2.1. Self-Assembly of Individual Nucleic Acids Bases at Vacuum

**Figure 1.**
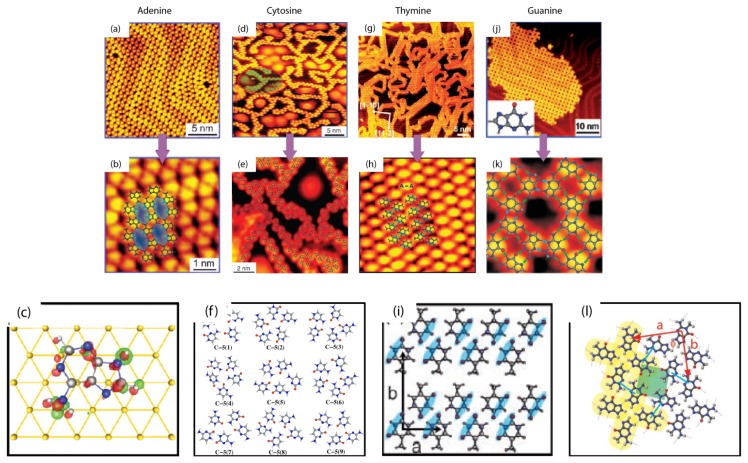
Experimental STM images and theoretical models for Adenine (**a**–**c**); Cytosine (**d**–**f**); Thymine (**g**–**i**); and Guanine (**j**–**l**) monolayers on the Au (111) surface. **a**, **d**, **g** and **j** show large areas of the experimentally observed DNA base monolayer structures, whereas **b**, **e**, **h** and **k** give close-up images with overlays of the theoretical models of Adenine, Cytosine, Thymine and Guanine, respectively. **c**, **f**, **i** and **l** show the molecular structures of these four kinds of single bases.

The basic components of NA bases are A: Adenine, C: Cytosine, T: Thymine and G: Guanine, which are generally capable of forming the specific hydrogen bonds following the rule of base pairing in the DNA molecule. It is therefore valuable to explore the self-assembly of NA bases A, C, T and G. In vacuum systems, the environmental effect is excluded and it is an ideal system for studying the assembled structures of NA bases. Adenine molecules deposited onto a clean Au (111) surface and self-assembled into well ordered 2D, apparently non-chiral islands, were recorded at 150 K (Figure 1a,b) [19]. In this honeycomb-like network each adenine molecule is connected with three neighbors. It can also be seen that the adsorption of molecules does not lift the herringbone reconstruction of the Au (111) surface, which indicates that the molecule-substrate interaction is so weak that the growth of the 2D self-assembled islands is mainly controlled by molecule-molecule interactions. Cytosine assembled structures can be significantly different with different surface coverage [20,21]. Here the blur indicates the nanocages. The filaments are always linked to some other filament through a 6-fold ring in a “roundabout” fashion, indicated with a red oval (Figure 1d–f). In addition, the “sticky ends” of any two filaments can join in a head-to-tail fashion, resulting in either bent or apparently linear filament structures. On the other hand, the “non-sticky” nature of the filament’s side borders make it difficult for molecules or molecular clusters trapped inside “nanocages” to attach to their “walls” and thus they diffuse fast in the enclosed regions (appeared as blurred protrusions in the STM images). Thymine molecules have the ability to self-assemble into hierarchical structures, step by step, from 1D filaments formed by hydrogen bonds to 2D islands formed via the weaker van der Waals interaction (Figure 1g–i) [22]. Initially, T molecules preferred to form filaments; with increasing deposited T molecules, some of the filaments assembled from T molecules start to attach to each other, side by side, resulting in some small patches of T islands. As the coverage is increased even further, well-ordered 2D T islands were the dominant structure on the surface. Guanine adsorbed on the Au (111) surface and self-assembled into guanine islands. Through STM study, it was found that the self-assembled G islands are composed of square building blocks (quartets) that are formed by four molecules. Inter- and intra-molecular hydrogen bonds dominate the formation of the G-quartets. These G-quartet structures were shown to be chiral [23,24].

The self-assemblies of NA bases at vacuum were explored and the assembled structures represent the assembled behavior of NA bases between themselves. The assembled structures of complementary bases and non-complementary bases are introduced in the next section.

### 2.2. Self-Assembly of Complementary and Non-Complementary Nucleic Acids Bases at Vacuum

**Figure 2.**
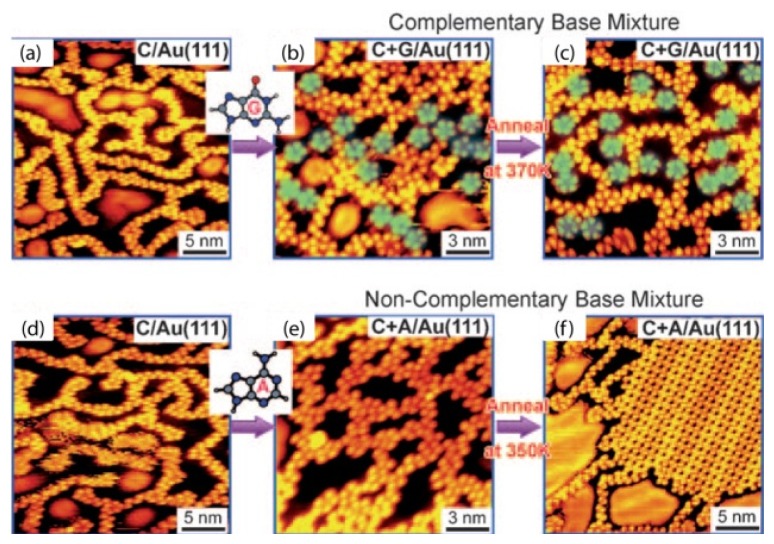
Co-deposition experiment for complementary C + G (**a**–**c**) and non-complementary C + A (**d**–**f**) bases. During the first step, similar amounts of C were deposited at room temperature in each case (**a**,**d**). After deposition of G (**b**) a sharp increase in the number of five-fold rings is found (indicated by the green shading), which was not observed after deposition of A (**e**). C + G and C + A mix on co-deposition (**b**,**e**). After heating, the complementary C + G mixture remains disordered (**c**), while the non-complementary C + A mixture segregates into A islands and C zigzag branches (**f**).

In the last section, we introduced the structures assembled by each of the four NA bases on a surface at vacuum. Here, the co-assembly of purine and pyrimidine will be introduced, such as the G–C complementary pair and the A–C non-complementary pair (Figure 2) [23]. Initially, G and A molecules are able to self-assemble into well-ordered 2D structures, while C molecules at lower coverage do not assemble into any structures, but form interconnected 1D zigzag filaments at high coverage. In the cases of co-assemblies of G–C and A–C, the different assembled behaviors were revealed, respectively. Pure C assembles were obtained initially, after the deposition of G and A molecules, the co-assemblies of G–C is obviously different from the ones of A–C. In the case of the co-assembly of G–C, some five-fold rings appear, but there is almost no change in the case of A–C assembly compared to the pure C assemblies. After annealing treatment, the assemblies of the G–C pair still exist, however, the assemblies of A–C segregated into two phases. A molecules form stable hexagonal 2D islands with C molecules present, but connecting with themselves in filaments and attaching to A island boundaries. It is suggested that the interaction between G and C must be much stronger than that in C–C and G–G pairs. Once the Watson-Crick complementary base pair is formed it will not break easily. However, the A–C pair is not as stable as the G–C pair, and it can easily be broken by thermal annealing. The binding energies for the most stable C–A pairs are between −0.75 and −0.88 eV, which are slightly smaller than, or comparable with, the most stable C–C (−0.99 eV) and A–A (−0.86 eV) pairs. Therefore, the segregation of A–C molecule assemblies is well explained.

The recognition and assembly process takes places in the absence of water; the NA bases are simply driven to self-assemble into structures by the energy difference between the hydrogen-bonded dimers, and temperature. The results obtained from the vacuum system fixes the minimum conditions under which a high fidelity, hydrogen bond-directed replication would be energetically advantageous. Therefore, it constitutes a minimum benchmark experiment compared to more realistic experiments at ambient condition, where many parameters such as conformational freedom, solvents and biochemical environment must be considered.

### 2.3. Self-Assembly of Individual Nucleic Acid Bases at Ambient Condition

**Figure 3.**
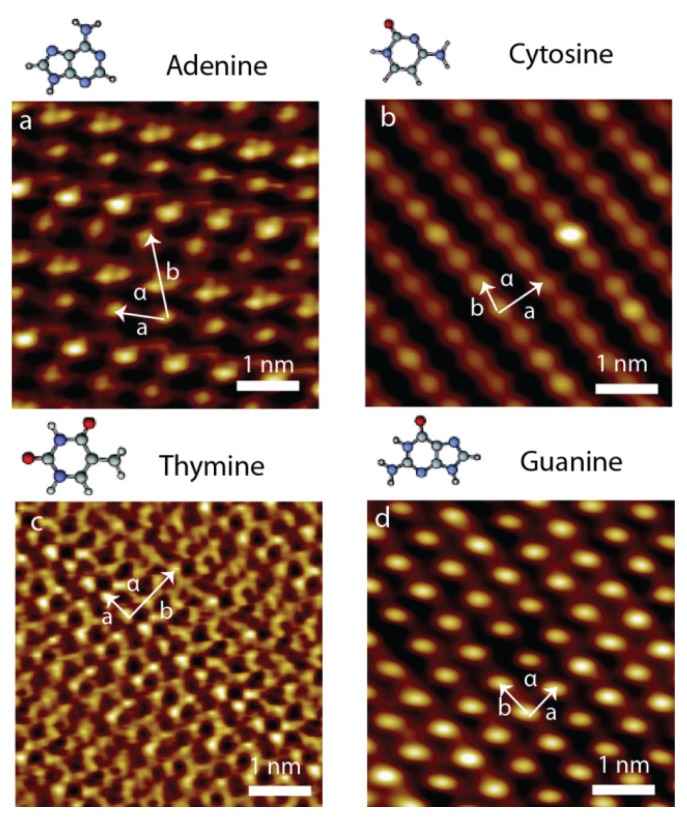
Typical STM images of pure NA bases adsorbed at the 1-octanol/HOPG interface. (**a**) Adenine; (**b**) Cytosine; (**c**) Thymine; and (**d**) Guanine. Scan size: 5 nm × 5 nm, *I*_set_ = 500–1000 pA, *V*_bias_ = 500–800 mV.

To pursue a more realistic environment for exploring the assembled behaviors of NA bases, the self-assembly of NA bases at ambient condition was investigated. Similar to the study at vacuum system, the assemblies of individual NA bases were explored first. The NA bases could also self-assemble into dimers by hydrogen bond formation at ambient condition. The gradual formation of well-ordered domains of NA base molecular assembly was revealed by STM imaging at the liquid/solid interface, which is closer to a realistic environment compared to vacuum systems (Figure 3). The well-ordered adlayers are displayed in the high-resolution images of individual NA base self-assemblies. Lattice parameters of observed different adlayer structures are summarized in Table 1. The adenine molecules could self-assemble into a stable 2D network arrangement with the determined unit cell *a* = 0.8 ± 0.1 nm, *b* = 2.2 ± 0.2 nm and α = 76.0° ± 2.3° (Figure 3a). The self-assembling structure of adenine molecules has also been reported on Ag-terminated Si (111) by hydrogen bond packing [7]. The cytosine molecules show the parallel chain alignment with a determined unit cell, where the lattice constants are *a* = 0.82 ± 0.08 nm, *b* = 0.53 ± 0.06 nm and α = 77.3° ± 2.3° (Figure 3b). The thymine molecules present the chain arrangement. The lattice constants include *a* = 0.8 ± 0.1 nm, *b* = 1.5 ± 0.2 nm and α = 87.0° ± 2.5° (Figure 3c). The chain arrangement was proposed by previous theoretical calculation [12]. The guanine molecules (Figure 3d) also present the ability to self-assemble into 2D network structures with a determined unit cell, where the lattice constants are *a* = 0.64 ± 0.07 nm, *b* = 0.68 ± 0.07 nm and α = 90.1° ± 2.6°.

There exist certain similarities in the 2D supramolecular structures observed for G and A, and similarly, between T and C. The 2D supramolecular networks based on G and A have similar hydrogen bonding dimer construction, whereas the parallel chain structure, constructed by T and C, involves the hydrogen bond dimer chains and van der Waals interaction between the chains. Thus, well-ordered assembling structures were constructed from pure NA bases at the liquid/solid interface. The network structures were commensurate with the energetically favorable network determined by the theoretical calculations. It is suggested from NA base assembling structures that NA dimers construct the base chain, and further, that the chains result in the formation of 2D networks by either inter-molecular hydrogen bonds or van der Waals interaction. The assembled behaviors of NA bases are different from the ones obtained at vacuum system. G-quartet formation obtained at vacuum does not exist at ambient condition. Some 1D filament structures assembled from cytosine and thymine molecules were not observed at ambient condition. The energetic advantages could not dominate the assembled structure forming at ambient condition, more factors were important, and finally NA bases prefer to self-assemble into dimer structures. An understanding of the properties of adsorbed NA bases at the liquid/solid interface is of high interest regarding both surface and synthesis. A variety of applications involving hydrogen bond mediated supramolecular chemistry and biotechnology could be based on NA base dimerization.

### 2.4. Self-Assembled Structure of Complementary Bases (G–C, A–T)

The designable and predictable DNA self-assembling nanostructures are achieved mainly due to using NA base sequences to encode instruction [1]. The complementary base pairing via hydrogen bonds in the double helical DNA structure is of utmost importance. The specific molecular interaction between DNA strands results in the precision and efficiency of its self-assembly into double helices and other fascinating nanostructures. Therefore, it is very significant and valuable to explore the assembling structure of pure NA bases by the specific hydrogen bond interaction. In the last section, we introduced the self-assembly of individual NA bases and reported the base network or base arrangement structures constructed by the four NA bases (A, C, T, G). In this section, we provide STM visualization of well-ordered assembled structures formed by co-adsorption of the complementary bases G–C and A–T at the liquid/solid interface.

#### 2.4.1. Self-Assembled Structure of Complementary Bases G–C

**Figure 4.**
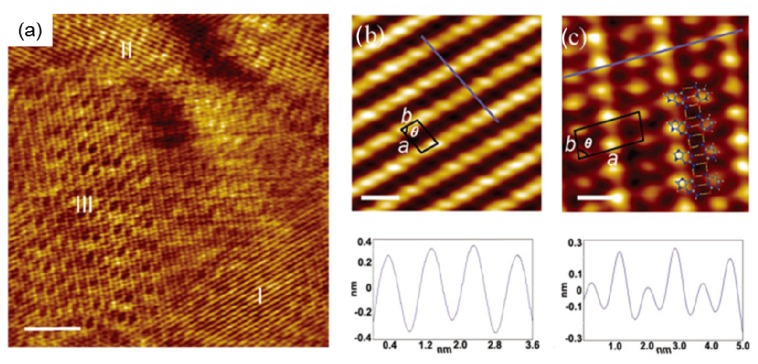
Co-adsorption of guanine and cytosine at the 1-octanol/HOPG interface. (**a**) Typical STM image presenting three domains, marked with I, II and III. Scan size: 50 nm × 50 nm; (**b**) Correlation averaged magnified image of the structure in domain I, 5 nm × 5 nm, a unit cell is indicated. There is a line profile on the bottom of (**b**) along the blue line in the top of the STM image; and (**c**) Correlation averaged magnified image of the structure in domain III, 5 nm × 5 nm, a unit cell is indicated. Bottom: height profile along the blue line in the top of panel. The structural model is superimposed on the STM image. *I*_set_ = 549 pA, *V*_bias_ = 750 mV. (Reproduced with the permission from [25]. Copyright 2006 American Chemistry Society.).

**Figure 5.**
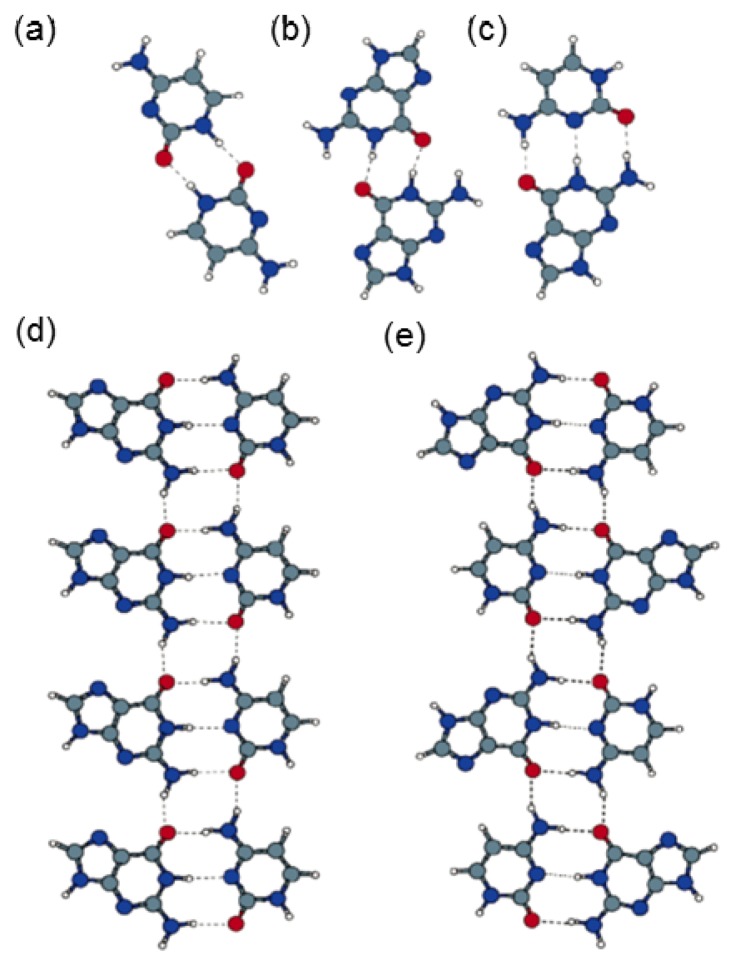
Structural model: (**a**) C dimer; (**b**) G dimer; (**c**) GC dimer; (**d**) Chain of GC dimers; and (**e**) Chain of GCGC-quartet formed by alternating GC dimers. (Reproduced with permission from [25]. Copyright 2006, American Chemistry Society.).

The hydrogen bond between guanine and cytosine is one kind of NA base complementary pairing. Guanine and cytosine are able to assembly into G-dimers and C-dimers, further forming G and C chains based on intermolecular hydrogen bonds between themselves. The co-adsorption of G and C on the surface will result in the co-assembly formed by G–C complementary pairing. The well-ordered adlayer is presented in the STM image (Figure 4 [25]). The formation of a distinct double row structure is clearly observed, and expected to be ascribed to aligned GC pairs. The co-assemblies of G and C on the surface are different from the structures assembled by pure G or C molecules. In the co-adsorption of G and C on the surface, three distinct domains of self-assembled structures can be identified, marked I, II and III (Figure 4a). The correlation averaging processing can improve the quality of high-resolution structures with molecular contrast. Domains I and II are composed of assembled bases with the feature aligned into straight and parallel rows. The periodicity of domain I/II is presented in Table 2. The parameter of the unit cell of domain I/II is determined to be *a* = 0.87 ± 0.09 nm, *b* = 0.45 ± 0.05 nm and θ = 77.3° ± 2.3°. The structure of domain I/II is in agreement with the structure formed by pure cytosine (Figure 3b). The lattice parameters of domain I/II are mostly similar to the ones of pure C assembling structures. It is therefore concluded that domain I and II are formed by cytosine molecule alone. Domain III, however, is in comparison, distinct from the previous STM images of pure C and G assemblies; it is indeed a mixed phase consisting of both C and G molecules. The periodic molecular arrangement alternates between rows of high and low protrusions within domain III (Figure 4c). G and C form heterodimers aligned into rows. The lattice parameters are determined to be *a* = 1.71 ± 0.18 nm, *b* = 0.69 ± 0.07 nm and θ = 84.1° ± 2.4°. To obtain an insight into the underlying molecule-molecule interaction, theoretical modeling based on the self-consistent charge density functional-based tight binding method was performed. It was determined that the planar structures for homodimers of G and C are the lowest energy configuration with binding energies of 0.4 (CC) and 0.5 eV (GG), presented in Figure 5a,b, respectively. The optimum heterodimer is definitely a Watson-Crisk GC pair (Figure 5c), which is found to have the considerably higher binding energy of 0.9 eV. It is therefore plausible that a GC dimer is the most preferential complex to form. GC dimers could also arrange into a row with an optimized model (Figure 5d). The periodicity along the model structure is 0.74 nm, consistent with the 0.69 ± 0.07 nm determined from the experiment, thus experiment and theoretical model are in good agreement. The row structure of GC dimers was stabilized in fact through the intermolecular hydrogen bonds. The dimer-dimer interaction energy values were obtained by calculating the binding energy per GC dimer. The binding energy for row segments with *n* GC dimers increase monotonically toward the limit for an infinite chain. It is the main reason explaining why the GC dimer could further assemble into the structures. It is also found that the so-called GCGC quartet was formed in four-stranded DNA quadruplexes due to the interaction between G and C [26]. The quartet structure can be realized from the model of Figure 5d, and by rotating the GC dimer by 180° around the surface normal each second, which results in the optimized structures shown in Figure 5e. The quartet row structure (G and C molecules alternate along the row direction) found from the calculation is slightly (0.1 eV per dimer) more stable than the dimer row observed by STM (Figure 4c), which appears to be inconsistent with the experiment. It is true that STM could not capture the configuration of GC pairs with the lowest binding energies, however, it also shows the possible assembling structures of G and C.

It is possible that the destabilizing coverage of the GC row structure in the experiment is mainly due to the absence of quartet chains. Furthermore, compared to the assembled structure of NA base pairs at vacuum, the assembly of NA bases formed at ambient condition was affected by more factors such as conformation freedom in the liquid interface and water solvent. The exploration of the adsorption and co-adsorption structures formed by NA bases guanine and cytosine at the liquid/solid interface by STM prove that hydrogen bonds between G and C, as well as between the GC dimers, will provide the main contribution to the formation of double row structures assembled by guanine and cytosine.

#### 2.4.2. Self-Assembled Structure of Complementary Bases T–A

**Figure 6.**
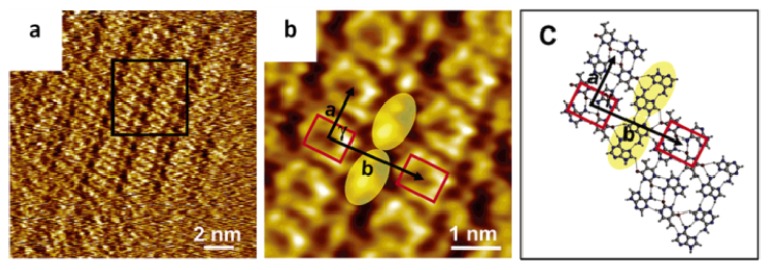
(**a**) STM image of A and T mixture at the 1-octanol/HOPG interface; (**b**) A correlation averaged zoom-in image of the part indicated in image (**a**) by a black square; and (**c**) The calculated model: each cycle is formed by four molecules 2A + 2T, resulting in reverse Hoogsteen A–T–A–T quartets adjacent to homochiral chains of A–A dimer. Cycle is indicated by red rectangles, and the unit cell is indicated in black. (Reproduced with permission from [27]. Copyright 2006 American Chemistry Society.).

Thymine and adenine are also capable of creating assembling structures on the liquid/solid interface, similar to the supramolecular structures assembled by G and C through hydrogen bond interaction. The hybrids formed by A and T have well-ordered molecular patterns that are significantly different from the assembled structures by pure adenine and thymine molecule (Figure 3c,d). In the mixture phase of A and T (Figure 6), there are two different coexisting features that are obviously revealed: (i) rows of cyclic structures (indicated with red rectangles) separated by (ii) single chains including only bright spots (indicated with yellow ovals) [27]. The pure A and T could not assemble into the structures with quartet cyclic feature, as observed in the mixed phase of A and T. In addition, the calculation proves that when four A molecules approach each other, dimer-A formation is favorable, and when two dimer-As bind together, the orientation of the A–A dimer will be altered from one row to another, which is shown in Figure 3a, leading to the network forming without the quartet cycles. Similarly, no T quartet cyclic structure was observed, and the calculation showed that T–T dimer formation is preferred over T quartet cyclic structures. It is therefore clear that the quartet cyclic structure is resulting from co-assemblies of A and T.

**Figure 7.**
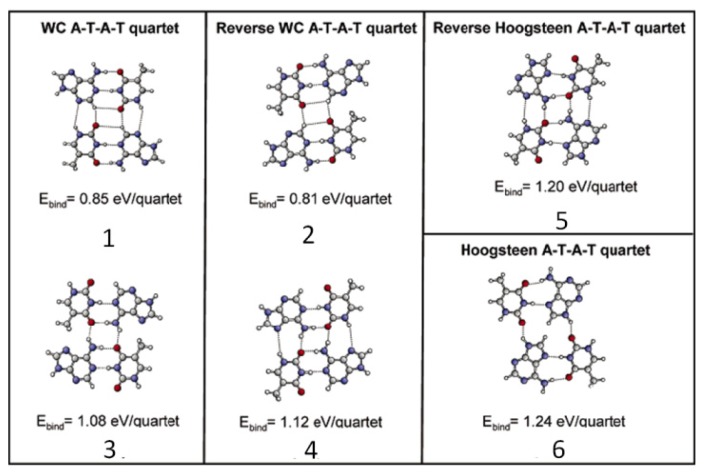
A–T–A–T quartets and their calculated binding energies. Numbers are used to identify the A–T–A–T quartets. (Reproduced with the permission from [27]. Copyright 2006 American Chemistry Society.).

When two mixed A–T heterodimers are close to each other, binding occurs between the A–T heterodimers resulting in the quartet cycle formation, which was observed by STM, and the unit cell of assembled structure is shown in Table 3. Due to the mixing molar ratio (350A:1T), more A molecules are involved in the assembly than T molecules that are mainly employed in the construction of A–T–A–T quartets. However, adenine molecules in A–A dimers are suggested to form the single chains that bind to the rows of A–T–A–T quartets to stabilize the whole network (Figure 6a,b). Furthermore, all kinds of quartet models in addition to that of single chain A-dimers were proposed (Figure 7) for identifying the most suitable model of the quartet, matching well with the molecular network assembled by A and T. Except for the reverse Hoogsteen A–T–A–T quartet model, all other calculated models were not reasonable and not suitable for the structures observed in STM. The reverse Hoogsteen [28] A–T–A–T quartets separated by homochiral chains of A–A dimers represent the best model according to: the contrast of the STM image; the size and shape of quartet cycles and single chains; and the binding energies between the quartets, as well as between the homo chiral chains of A–A dimers. The binding energy of this most stable overall molecular network is 2.03 eV (per one A–T–A–T quartet + one A–A dimer in the unit cell). The unit cell parameters from calculations is determined to be *a* = 1.0 ± 0.2 nm, *b* = 2.3 ± 0.2 nm and γ = 88.0° ± 2.0° which is consistent with STM findings. Furthermore, in the reverse Hoogsteen quartet, eight hydrogen bonds exist, whereas there are only two hydrogen bonds between two reverse Hoogsteen A–T–A–T quartets along the unit cell vector a (Figure 6c).

Generally, combining the STM study and the theoretical calculations, the co-assembled structures by adenine and thymine molecules were explored. The reverse Hoogsteen A–T–A–T quartets separated by homochiral chains of A-dimers were constructed initially; they subsequently self-assembled into an overall stabilization of a molecular network. The cyclic features formed by the A and T mixture are closely correlated to the so-called quardruplex strucures that consist of only one type of NA bases, or a mixture of complementary DNA bases, which is found in biological system processes such as replication, transcription, recombination and telomere function [29].

## 3. Conclusions and Perspectives

We have presented novel 2D supramolecular nanostructures assembled by NA complementary bases on a surface. NA bases have the versatility to self-assemble into a wide variety of nanoscale patterned structures. Pure guanine and adenine are able to assemble into molecular network patterns on the surface by hydrogen bond interaction; cytosine and thymine are capable of constructing the molecular chain structure. However, the self-assembled nanostructures formed by the mixture of G–C and A–T are significantly different from the ones formed by pure DNA bases. In regard to the supramolecular chemistry, these specific interactions from the NA bases could make the field very intriguing due to the wealth of supramolecular bonds that NA bases use to interact with each other [30]. There are 28 possible base pairing motifs, employing at least two hydrogen bonds, formed between these four NA bases [4]. The placement of these NA bases on the end of small molecules or polymers, when introducing the specific interaction, will help to construct a variety of supramolecular nanostructures that can be applied in either biological or material systems [30]. To obtain insight into the self-assembled behaviors of these supramolecular systems, STM studies at ambient condition will provide a potential approach towards visualizing the assembled structures on the surface. The observation and understanding of these novel nanoscale patterns formed from NA base pairing based systems will not only be interesting from the perspective of molecular organization at interfaces, but it will be useful for understanding the mechanism of material construction [30]. Furthermore, the study of self-assemblies of nucleic acid base molecules is of great use for the future understanding of the mechanisms behind the assemblies of NA bases and their complexes with amino acids and protein or more complex biological systems.

## Figures and Tables

**Table 1.** Comparison of the lattice parameters of the observed different assembly structures of NA bases as derived from the analysis of the STM images.

	*a* (nm)	*b* (nm)	α (degree)
**Pure G**	0.64 ± 0.07	0.68 ± 0.07	90.1 ± 2.6
**Pure C**	0.82 ± 0.08	0.53 ± 0.06	77.3 ± 2.3
**Pure A**	0.80 ± 0.10	2.20 ± 0.20	76.0 ± 2.3
**Pure T**	0.80 ± 0.10	1.50 ± 0.20	87.0 ± 2.5

**Table 2.** Comparison of lattice parameters for the different assembly structures of G, C and G–C from the analysis of STM images. (Reproduced with permission from [25]. Copyright 2006 American Chemistry Society.).

	*a* (nm)	*b* (nm)	θ (degree)
**Domain I and II**	0.87 ± 0.09	0.45 ± 0.05	76.7 ± 2.2
**Domain III**	1.71 ± 0.18	0.69 ± 0.07	84.1 ± 2.4
**Pure cytosine**	0.82 ± 0.08	0.53 ± 0.06	77.3 ± 2.3
**Pure guanine**	0.64 ± 0.07	0.68 ± 0.07	90.1 ± 2.6

**Table 3.** Comparison of lattice parameters for the different assembly structures of A, T and A–T from the analysis of STM images. (Reproduced with the permission from [27]. Copyright 2006 American Chemistry Society.).

	*a* (nm)	*b* (nm)	γ (degree)
**Pure adenine (A)**	0.8 ± 0.1	2.2 ± 0.2	76.0 ± 2.3
**Pure thymine (T)**	0.8 ± 0.1	1.5 ± 0.2	87.0 ± 2.5
**Mixture A + T**	1.0 ± 0.2	2.3 ± 0.2	88.0 ± 2.0
